# Pamidronate Disodium for Palliative Therapy of Feline Bone-Invasive Tumors

**DOI:** 10.1155/2014/675172

**Published:** 2014-06-09

**Authors:** Jackie M. Wypij, David A. Heller

**Affiliations:** ^1^University of Illinois at Urbana-Champaign, Urbana, IL 61802, USA; ^2^Advanced Veterinary Care Center, Lawndale, CA 90260, USA

## Abstract

This study sought to quantify *in vitro* antiproliferative effects of pamidronate in feline cancer cells and assess feasibility of use of pamidronate in cats by assessing short-term toxicity and dosing schedule in cats with bone-invasive cancer. A retrospective pilot study included eight cats with bone invasive cancer treated with intravenous pamidronate. *In vitro*, pamidronate reduced proliferation in feline cancer cells (*P* < 0.05). One cat treated with pamidronate in combination with chemotherapy and two cats treated with pamidronate as a single agent after failing prior therapy had subjective clinically stable disease; median progression free interval in these cats from initial pamidronate treatment was 81 days. Three cats developed azotemia while undergoing various treatment modalities including nonsteroidal anti-inflammatory drugs and pamidronate. Median overall survival was 116.5 days for all cats and 170 days for cats with oral squamous cell carcinoma. Median progression free survival was 55 days for all cats and 71 days for cats with oral squamous cell carcinoma. Pamidronate therapy appears feasible for administration in cancer bearing cats with aggressive bone lesions in the dose range of 1-2 mg/kg every 21–28 days for multiple treatments. No acute or short-term toxicity was directly attributable to pamidronate.

## 1. Introduction

Primary bone cancer such as osteosarcoma is a common problem in dogs but rare in cats [1, 2]. Cats are more likely to suffer from secondary malignant bone lesions such as locally invasive soft tissue cancers or metastatic lesions with oral squamous cell carcinoma (OSCC), the most common bone-invasive tumor [3, 4]. Many advanced tumors are unresectable resulting in malignant osteolysis and bone pain. In people, bone cancer causes pain, hypercalcemia, anemia, susceptibility to infection, skeletal fractures, and decreased mobility, all of which compromise patient functional status, quality of life, and survival [5, 6]. Although quality of life can be difficult to assess in feline patients, bone-invasive tumors may also compromise functional status, quality of life, and survival in cats.

Optimal treatment of feline cancer-related bone pain remains elusive. Conventional treatments include surgical removal, external beam radiation therapy, and oral analgesics. While limb amputation results in good outcomes, surgery is often not feasible for other advanced bone-invasive tumors. Radiation therapy can be very effective with advancements in delivery and patient positioning improving outcome. Radiation therapy may be limited by geographic accessibility, financial cost, necessity of repeat sedation/anesthesia, and adverse local tissue side effects and is not directly available to the clinical practitioner. Oral analgesic drugs are limited by species-specific toxicity, efficacy, and client's ease of administration, particularly in cats with primary oral tumors. Chronic cancer pain remains difficult to be addressed with oral analgesics and no nonsteroidal anti-inflammatory drugs (NSAIDs) are currently approved for long-term use in cats [7, 8]. These factors may result in inadequate management of feline bone cancer pain necessitating novel alternative options.

Bisphosphonates are bone-remodeling drugs used to treat malignant bone lesions and hypercalcemia of malignancy. Pamidronate (Aredia, Novartis Pharmaceuticals Corporation, East Hanover, New Jersey, USA) and zoledronate (Zometa, Novartis Pharmaceuticals Corporation, East Hanover, New Jersey, USA) are aminobisphosphonates (NBPs) considered standard of care therapy for nonresectable skeletal metastases in human cancer patients [9]. Pamidronate provides cost-effective pain relief with comparable outcomes to zoledronate [10, 11]. Although the primary effect of NBPs appears to be osteoclast inhibition resulting in modulation of bone pain and skeletal fractures, some NBPs including zoledronate and pamidronate also possess direct antineoplastic effects [12].

In dogs, monthly parenteral administration of pamidronate or zoledronate is modestly effective in palliating bone cancer pain for osteosarcoma and other bone tumors [13–15] but does not appear to improve survival [16, 17]. Clinical bisphosphonate use in cats has been limited to case reports of non-cancer-related hypercalcemia and ossifying myositis [18–20]. In a prospective study, zoledronate reduced serum biomarkers of angiogenesis (vascular endothelial growth factor/VEGF) and bone turnover (C-terminal telopeptide/CTx) in cats with oral squamous cell carcinoma, suggesting effects on malignant osteolysis as well as indirect anti-cancer effects [21]. Clinical application of zoledronate (Zometa, Novartis Pharmaceuticals Corporation, East Hanover, New Jersey, USA) which is commercially available in individual vials appropriate for a 40 kg dog may be limited by cost in smaller veterinary patients.

Pamidronate may be useful in managing chronic pain associated with cancer and may possess direct or indirect anticancer effect in feline tumors. To date, no studies have evaluated the effectiveness of bisphosphonates for pain control or direct tumor effects and no studies have evaluated pamidronate in cancer-bearing cats. We hypothesize that pamidronate is a feasible adjuvant therapy for cats with bone-invasive tumors with minimal toxicity and simple intermittent administration. The aims of this study were to (1) investigate* in vitro* effects of pamidronate on proliferation in two cells lines representative of feline bone-invasive cancer and (2) assess the feasibility, short-term toxicity, putative dose range, and clinical response in cats with bone-invasive cancer treated with pamidronate in combination with other therapies.

## 2. Materials and Methods

### 2.1. Cell Lines and Reagents

A feline OSCC cell line (SCCF1, provided by Dr. Thomas J. Rosol, The Ohio State University) was grown in Williams E media supplemented with 2 mM l-glutamine, 0.05 mg/mL gentamicin, 10 ng/mL epidermal growth factor, 0.01 nM cholera toxin, and 10% fetal bovine serum (FBS). A feline fibrosarcoma (FSA) cell line (FC83, purchased from ATCC) was grown in DMEM supplemented with 10% FBS. Cell cultures were maintained in subconfluent monolayers at 37°C in a 5% CO_2_ humidified chamber and passaged twice weekly. Pamidronate was purchased from Sigma-Aldrich (St. Louis, MO) and stock solutions (1 mg/mL) were prepared in sterile phosphate-buffered saline (PBS), aliquoted, and frozen at −20°C until use. Cells were cultured in media as described above for controls; no vehicle control was necessary given use of PBS as pamidronate drug vehicle.

### 2.2. *In Vitro* Cell Proliferation

Cells were seeded in a 96-well plate overnight at 5 × 10^3^ cells/well. Cells were treated with 0–100 *μ*M pamidronate and extrapolated from previously published canine data [22, 23] encompassing the range of the putative peak plasma concentration of 9.7 *μ*M [24, 25]. Cell proliferation was assessed after 24 hr of pamidronate treatment using manufacturer's recommended protocol with a commercial assay (CellTiter 96^®^ AQueous One Solution MTS, Promega, Madison, WI, USA) that quantitates metabolically active viable cells using a colorimetric tetrazolium salt. The experiment was performed in quadruplicate with repetition. Media control was set as 100% proliferation with experimental groups expressed as percent of control.

### 2.3. Retrospective Pilot Study

A retrospective study was performed of client-owned cats diagnosed with bone-invasive tumors. Medical records were reviewed for cats presented to the oncology services of the University of Illinois at Urbana-Champaign and Advanced Veterinary Care Center during the time period 2005–2012. Inclusion criteria were (1) cats with bone-invasive cancer based on physical examination and imaging, (2) pamidronate therapy, (3) minimum follow-up of three- weeks after treatment, and (4) pre- and postpamidronate serum renal profiles including creatinine, blood urea nitrogen, calcium, phosphorus, and potassium. Exclusion criteria were (1) life-limiting comorbidities, (2) incomplete medical records, or (3) inadequate follow-up.

Clinical data extracted from the medical records included signalment, weight, duration and type of presenting clinical signs, method of diagnosis, clinical stage, clinicopathologic data, adverse side effects, clinical response, cause of death, and survival. Contemporary standard of care treatment was offered to each client. Treatment information included previous, concurrent, and subsequent surgery, chemotherapy, and radiation therapy, as well as supportive medications. Full clinical staging was not performed on all cats either due to advanced disease or client decision. At each visit a subjective assessment was made based on physical examination, body condition/weight, physical function of affected area, and standard hospital pain score (0–10) combined with owner's observations as documented on a questionnaire at each visit. Subjective assessment was used to ensure adequate patient comfort and nutritional status was maintained regardless of objective measurements. Objective clinical tumor response was reported based on caliper measurement of primary tumor using World Health Organization (WHO) criteria: complete response, CR (disappearance of all known disease), partial response, PR (≥50% decrease from baseline), stable disease, SD (<50% decrease or <25% increase from baseline) and progressive disease, and PD (≥25% increase from baseline or new lesions). The minimum time interval used to determine objective tumor response was three weeks, which was considered appropriate given the aggressive nature of the tumor types, presence of bone invasion, advanced stage of disease, and prior failure of standard therapy in four of the cats. Treatment failure was defined as progressive disease as assessed at 3-4-week intervals based on WHO criteria. Patients with progressive disease during/immediately after radiation therapy were considered to have failed radiation despite the theoretical potential for delayed tumor cell kill. Given the retrospective nature and small sample size, primary outcome objectives were limited to (1) feasibility and practicality of administering treatment to cats in a clinical setting and (2) acute and short-term adverse side effects (hematologic, biochemical, and gastrointestinal) attributable to pamidronate therapy. Secondary outcome objectives were evaluation of clinical response, progression free interval calculated from first pamidronate treatment, and overall survival calculated from date of diagnosis. Subjective pain control was recorded; however, the direct effects of pamidronate on modification of pain in this population could not be assessed given the retrospective nature, multiple clinician observers, difficulty of objectively assessing pain in cancer-bearing cats, and multimodality treatment including various oral analgesics and radiation.

### 2.4. Statistical Analysis

Distribution of the data was assessed graphically as the data sets were considered too small for the use of a statistical normality test. Normal distributed data sets were expressed as mean ± standard deviation, and nonnormal distributed data sets were expressed as median and range. A one-way ANOVA was performed to assess the dose-dependent biologic activity of pamidronate in cell lines with post hoc Dunnett's test. Analysis of survival was performed with Kaplan-Meier survival curve with no animals censored. Statistical analysis was performed using a commercial computer software suite (GraphPad Instat3, Prism5, Statmate) with *P* < 0.05.

## 3. Results 

### 3.1. *In Vitro* Cell Proliferation

**Figure 1:**
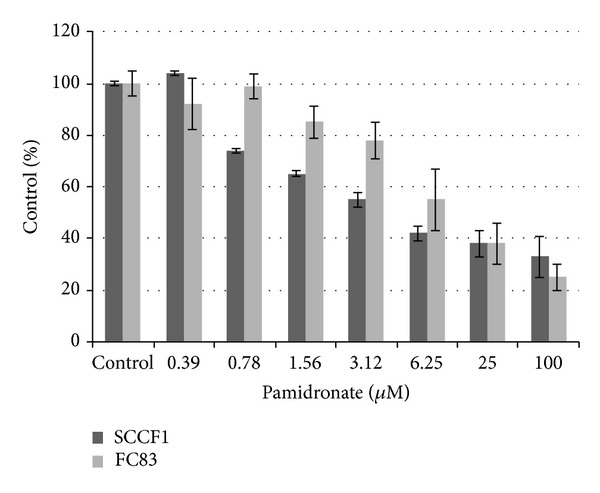
Pamidronate decreases cell proliferation in a dose-dependent manner. For doses ≥6.25 *μ*M for both cell lines, *P* < 0.05 compared to untreated control. Error bars represent SD.

In an effort to quantify direct antineoplastic effects of pamidronate on representative cell lines with bone-invasive potential, we evaluated effect on cell proliferation in feline OSCC and FSA. Pamidronate decreased* in vitro* cell proliferation in a dose-dependent manner with an IC50 of 4.2 *μ*M in SCCF1 cells and 15.1 *μ*M in FC83 cells (Figure 1). Reduction in cell proliferation was significant for concentrations ≥0.78 (SCCF1) and ≥6.25 *μ*M (FC83) (*P* value between *P* < 0.05 and *P* < 0.001).

### 3.2. Retrospective Pilot Study

Eight cats met the inclusion criteria and were included in the study. Patient characteristics are summarized in Table 1.

Six cats were diagnosed via histopathology and two cats via cytopathology. Treatment details are summarized in Table 2.

Surgical treatment was not deemed feasible in any cat. Tumors in all six cats with squamous cell carcinoma (SCC) were large and considered unresectable based on physical examination and/or CT scan (performed in 3/6 cats). Surgery was not recommended for the cat with pulmonary carcinoma given the metastatic disease. Surgery was declined by the owner (limb amputation) of the cat with osteosarcoma due to perceived quality of life concerns. Additional staging at diagnosis consisted of radiographs, abdominal ultrasound, blood work, urinalysis, and lymph node cytology. Three view thoracic radiographs (7/8 cats), abdominal ultrasound (4/8 cats), and regional lymph node cytology (3/8 cats) were performed with no evidence of metastasis. All cats had initial complete blood count and serum chemistry followed by serial blood work monitoring, including a renal profile or general chemistry profile immediately prior to and 1–3 weeks after pamidronate treatment. Urinalysis was available in all cats prior to treatment; however, serial urine specific gravity was not available in all cats concurrent with biochemistry during treatment. All cats received some form of chemotherapy and/or radiation therapy prior to (four cats) or concurrently with (four cats) pamidronate treatment. Patients undergoing radiation therapy received a dose of 8 Gy weekly for three to four consecutive weeks using Cobalt-60. Cats were sedated for radiation therapy with a combination of dexmedetomidine, glycopyrrolate, and butorphanol intramuscularly. All cats received chronic oral analgesics including one or more of piroxicam, meloxicam, tramadol, buprenorphine, butorphanol, and gabapentin

For clinical administration, pamidronate was reconstituted in sterile water per manufacturer's recommendations and diluted into 40–60 mL 0.9% saline. An indwelling peripheral intravenous catheter was placed in all cats with pamidronate administered at 1-2 mg/kg (median 1.2 mg/kg) as a constant rate infusion over two hours every 21–28 days. Median cumulative pamidronate dose was 8.5 mg (range 2.6–29.2 mg). No catheter complications or local tissue irritation were noted. No cats required sedation for pamidronate administration; however, two cats were receiving sedation for concurrent radiation therapy. Patients were evaluated every 21–28 days unless receiving concurrent radiation therapy (evaluated weekly). No cats were lost to follow-up.

No patient achieved partial or complete remission regardless of treatment. Four cats with prior treatment failure defined as tumor progression (three radiation, one radiation and chemotherapy) were treated with pamidronate as a single agent. Using the WHO criteria described previously, of these four cats, two cats had clinically stable disease for greater than three weeks and two had progressive disease. Four cats without any prior therapy were treated with pamidronate as adjuvant therapy, with concurrent radiation (1 cat), chemotherapy (1 cat), or radiation and chemotherapy (2 cats). Of these four cats, one had stable disease (concurrent chemotherapy) and three had progressive disease despite concurrent chemotherapy and/or radiation. Overall, three of eight cats had subjective clinical stabilization of disease and received multiple (2–4) doses of pamidronate with a median progression free interval of 85 days from start of pamidronate therapy (range 71–86 days). Of these three cats, one cat was treated concurrently (mitoxantrone) while two cats were treated with single-agent pamidronate after previously failing radiation (1 cat) or radiation/chemotherapy (1 cat). The cat treated with concurrent mitoxantrone and pamidronate received subsequent chemotherapy including carboplatin, gemcitabine, and toceranib phosphate (Palladia, Pfizer Animal Health, New York, NY, USA). Dose of pamidronate could not be correlated to response given the variability in treatments and small sample size.

Although gastrointestinal toxicity is rare, one potential acute or short-term hematologic sequela to pamidronate treatment is anemia and thus clinical monitoring parameters in human cancer patients include complete blood counts [25]. No cats receiving pamidronate alone or concurrently with radiation therapy had adverse gastrointestinal or hematologic side effects. All three cats receiving chemotherapy concurrently with pamidronate developed adverse side effects. Two cats (concurrent mitoxantrone) developed grade 1-2 gastrointestinal side effects, and one cat (concurrent carboplatin) developed grade 3 nonfebrile neutropenia [26]. All cats were treated with supportive care with resolution of clinical signs. No subsequent dose reductions or treatment delays were necessary.

Potential acute or short-term biochemical sequelae to pamidronate treatment include azotemia and electrolyte abnormalities (hypocalcemia, hypokalemia, and hypophosphatemia) and thus clinical monitoring parameters in human cancer patients also include creatinine and electrolytes [25]. While all cats had serial serum renal profiling prior to and following each pamidronate treatment, urinalyses were not serially measured in all cats. Pre- and postpamidronate serum ALT were available in 4/8 cats with no evidence of elevation. Serum electrolytes were available in all cats with no evidence of hypocalcemia, hypophosphatemia, or hypokalemia. The cat with chronic renal insufficiency (IRIS stage 2) did not develop worsened azotemia. Three cats with no pretreatment evidence of kidney disease developed mild to severe azotemia at various times while receiving multiple therapies including pamidronate (Table 3).

Median change after pamidronate treatment in serum creatinine of all cats was 0.2 mg/dL (range: 0.2 to 5.3) and median change in BUN was 5.5 mg/dL (range: 12 to 181). Correlation of azotemia with pamidronate or other treatments could not be evaluated given the small sample size and multiple potential nephrotoxic treatment factors including concurrent sedative use, concurrent NSAID use, and concurrent chemotherapy (Table 4).

Median overall survival of all cats was 116.5 days (range 24–247 days) and median overall survival of cats with oral SCC was 170 days (range 43–189 days). Median progression free survival of all cats was 55 days (range 21–86 days) and median progression free survival of cats with oral SCC was 71 days (range 21–86). All cats were followed until death and no necropsies were performed. Seven cats were euthanized due to clinically progressive cancer and one was euthanized due to renal failure.

## 4. Discussion

Pamidronate reduces skeletal morbidity, delays tumor progression, and improves overall survival in people with malignant bone lesions [9, 12]. Pamidronate's* in vitro* effects include reduction of proliferation, induction of apoptosis, inhibition of angiogenesis, and immunostimulation [12]. In human cancer patients, a standard intravenous dose of 1 mg/kg results in a peak plasma concentration of 9.7 *μ*M. The dose used in our patients was extrapolated from that published in people, dogs, and cats (1-2 mg/kg) [13, 15, 18–20, 25]. In this study of neoplastic feline cell lines with bone-invasive potential [3, 4], pamidronate demonstrated direct* in vitro* anticancer activity. Feline cells (SCC and FSA) demonstrate comparable sensitivity to canine OSA cell lines [22, 23] with antiproliferative effects within the putative achievable serum concentration (9.7 *μ*M). While* in vitro* evaluation in both canine and feline cell lines remains limited and comparison across tumor types is difficult, our data suggests that pamidronate may have a similar response in cancer-bearing cats to the modest clinical results reported in dogs and supports the biologic rationale to administer pamidronate to cancer-bearing cats.

Pamidronate therapy has not previously been described in cancer-bearing cats; however, single dose intravenous pamidronate has been used to treat non-cancer-related hypercalcemia in three cats [18, 19]. One cat with idiopathic hypercalcemia developed moderate ionized hypocalcemia and worsening hypophosphatemia after pamidronate treatment [18]. One cat with nocardiosis developed mild reduction in total serum calcium but maintained normal ionized levels, and one cat with idiopathic hypercalcemia and chronic renal failure did not develop abnormal electrolyte changes [19]. Hypercalcemia was not a factor in our patient population, and no clinically significant reductions in calcium, phosphorus, or potassium were documented after pamidronate treatment in this population; however, feline hypercalcemia of malignancy related to malignant osteolysis may also represent a therapeutic target for pamidronate.

We sought to assess the feasibility, short-term toxicity, putative dose range, and clinical response in cats with bone-invasive cancer treated with pamidronate in combination with other therapies. Our first primary objective confirmed the practicality of administering treatment to cats in a clinical setting. Common reactions in human cancer patients at the infusion site of pamidronate include pain, redness, swelling, induration, phlebitis, and thrombophlebitis [25]. All cats in this study tolerated the intravenous catheter and infusion without issues and no local reactions were documented. Unlike conventional chemotherapy agents, pamidronate is not mutagenic or carcinogenic [27, 28]. Nitrile, latex, or rubber gloves are recommended to limit mild skin irritation; however, other conventional personal protective equipment is not required during routine therapeutic administration, making this drug practical in general veterinary practice [25, 27, 28].

Our second primary objective was to assess acute and short-term adverse side effects (hematologic, biochemical, and gastrointestinal) attributable to pamidronate therapy. In human patients, pamidronate does not appear to increase toxicity associated with conventional chemotherapy administration [29]. Of cats receiving chemotherapy in our study, three cats exhibited gastrointestinal or hematologic toxicity that was clinically attributed to conventional chemotherapy. While the potential contribution of pamidronate to the toxicity observed in these cats cannot be ruled out, the mild to moderate clinical signs remain clinically acceptable. In human cancer patients, a major potential adverse effect of bisphosphonate therapy is nephrotoxicity [30–32]. Nephrotoxicity is less common with pamidronate compared to zoledronate with therapeutic use in people and can be minimized with diuresis, monitoring of serum creatinine, drug holiday with transient renal insufficiency, and dose adjustment in patients with preexisting kidney disease [33]. In preclinical studies, cats and dogs treated with pamidronate at 2–20 mg/kg/week for three months developed azotemia and tubular degeneration and necrosis [27]. In our study, one cat with preexisting renal insufficiency did not worsen, yet three of the initially nonazotemic cats developed renal disease. In this small group of patients, all cats that developed azotemia also received prior sedation and concurrent meloxicam. The cat treated at the highest dose in this study (2 mg/kg) also received the highest cumulative dose (29.2 mg) and did not develop azotemia despite prior sedation and concurrent piroxicam treatment. Of particular concern, meloxicam was approved for chronic use in cats in the United States and most of the cats in this study were treated prior to the 2010 “black box” warning from the FDA regarding potential for acute renal failure and death in cats [8, 34].

Our secondary objective was to describe clinical response, progression free interval from first pamidronate treatment, and overall survival time. Although multimodality treatment and retrospective nature preclude any conclusions regarding the efficacy of pamidronate therapy for palliation of bone pain or anticancer effects, we identified three cats with a subjective clinical response durable for >70 days, including two cats that had tumor progression following conventional radiation therapy. Although the time to maximum effective pain relief in dogs with bone tumors supports a delayed effect of radiation, these cats were considered to have failed radiation therapy given objective tumor size progression. However, subjective bone pain relief may be attributable prior to concurrent therapy although this was not directly assessed in the cats. These modest results remain encouraging as all cats had advanced disease and minimal response to conventional therapies.

Inherent limitations of the* in vitro* study include ability to extrapolate from cancer cell types to clinical patients as well as lack of information regarding the underlying drug mechanism of action. Limitations of this retrospective pilot study include selection bias, lack of controls, small number of animals, short follow-up period, and use of concomitant therapies.

## 5. Conclusions

Our study provides a biologic rationale for anticancer effects of pamidronate in feline tumors. To our knowledge, this is the first study demonstrating* in vitro* activity of pamidronate in feline cancer cells and the first study documenting repeat administration of pamidronate and evaluating toxicity in cancer-bearing cats. We demonstrate that pamidronate therapy is feasible for administration in cancer bearing cats with advanced neoplastic bone lesions in the dose range of 1-2 mg/kg every 21–28 days for multiple treatments and is practical for administration in general practice. No acute or short-term toxicity (hematologic, biochemical, or gastrointestinal) could be directly attributed to pamidronate, although the multimodal therapy in these patients may obscure potential toxic effects of pamidronate. The advanced nature of the cancers precluded long-term evaluation. Subjective clinical response and pain control were documented in individual patients but are likely reflective of the multimodality therapy and thus we cannot draw any conclusions regarding the efficacy of pamidronate in either palliation of bone pain or anticancer efficacy. Potential benefits of pamidronate in combination with conventional therapy include minimal costs and in-clinic administration of a long-acting putative adjuvant analgesic without the client compliance issues of at-home oral medications. In addition to the tumors treated here, (OSCC, aural SCC, osteosarcoma and rib metastasis of pulmonary carcinoma), other tumor histologies with bone-invasive potential that may benefit include soft tissue sarcomas such as vaccine-associated sarcomas and fibrosarcoma, multiple myeloma, and tumors of the orbit, digit, ear canal, vertebra, and sinonasal cavity. [4, 35–45]. Caution should be taken when administering pamidronate with preexisting renal disease or in conjunction with other potentially nephrotoxic drugs. A prospective clinical trial of pamidronate is warranted to further investigate chronic toxicity and potential effectiveness in palliating bone pain and limiting tumor progression in cancer-bearing cats.

## Figures and Tables

**Table 1:** Patient characteristics.

Age	Median 11 y
	Range 8–15 y

Sex	3 FS
	5 MC

Breed	7 DSH
	1 Siamese

Weight	Median 5.05 kg
	Range 2.93–7.5 kg

Tumor type/location	6 SCC
	2 mandible
	3 maxilla/palate
	1 aural
	1 rib metastasis
	(pulmonary carcinoma)
	1 osteosarcoma (ulna)

Prior versus concurrent therapy	6/8 radiation therapy
	4/6 prior to pamidronate
	2/6 concurrent
	4/8 chemotherapy
	1/4 prior to pamidronate
	3/4 concurrent

**Table 2:** Treatment details in cats receiving pamidronate.

Cat	Diagnosis	Treatment prior to pamidronate (failed)	Treatment concurrent with pamidronate	Pamidronate dose and number of doses	Response to pamidronate +/− concurrent therapy	Overall survival time (days)
1	Aural SCC	Radiation therapy, 24 Gy		1 mg/kg, number 1	PD	24
2	Oral SCC	Radiation therapy, 24 Gy		1 mg/kg, number 3	SD, 85 d PFI	170
3	Oral SCC	None	Radiation therapy, 24 Gy	1.5 mg/kg, number 1	PD	43
4	Oral SCC	Radiation therapy, 32 Gy mitoxantrone		2 mg/kg, number 4	SD, 86 d PFI	189
5	Pulmonary carcinoma rib metastasis	None	Carboplatin	1 mg/kg, number 1	PD	63
6	Oral SCC	None	Mitoxantrone	1.5 mg/kg, number 1	PD	47
7	OSA	Radiation therapy, 32 Gy		1 mg/kg, number 1	PD	247
8	Oral SCC	None	Mitoxantrone	1.4 mg/kg, number 2	SD, 71 d PFI	180

**Table 3:** Development of azotemia in relation to pamidronate treatment.

Cat	Azotemia relative to first dose of pamidronate	Creatinine (range 0–1.5 mg/dL)		BUN (range 14–34 mg/dL)	
		Initial	First episode azotemia	Initial	First episode azotemia
1	2 weeks post	1.1	2.5	15.9	38.4
2	20 weeks post	1.5	6.8	29.3	181
3	6 weeks post	1.5	4.4	34	52

**Table 4:** Potential treatment factors contributing to azotemia.

Cat	Azotemia relative to initial pamidronate	Potential nephrotoxic factors			
		Number of pamidronate treatments	Cumulative pamidronate mg/kg	Concurrent NSAID	Sedation or chemotherapy concurrent (C) with or prior (P) to azotemia
1	2 weeks post	1	1	Meloxicam	Sedation (P)
2	20 weeks post	3	3	Meloxicam	Sedation (P)
3	6 weeks post	1	1.5	Meloxicam	Sedation (P)
4	None	4	8	Piroxicam	Sedation, carboplatin, mitoxantrone (P)
5	None	1	1	Piroxicam	Carboplatin (C)
6	None	1	1.5	None	Sedation, mitoxantrone (C)
7	Stable CRF	1	1	None	Sedation (P)
8	None	2	2.8	Meloxicam	Mitoxantrone, carboplatin, gemcitabine, Palladia (C)

## Abbreviations

OSCC: Oral squamous cell carcinoma
SCC: Squamous cell carcinoma
NBP: Aminobisphosphonate.
